# Constructed growth charts and nutrition for pontocerebellar hypoplasia type 2A

**DOI:** 10.1111/dmcn.16394

**Published:** 2025-07-15

**Authors:** Alice Kuhn, Maren Hackenberg, Anna‐Lena Klauser, Antonia Herrmann, Julia Matilainen, Simone Mayer, Saskia Frölich, Ingeborg Krägeloh‐Mann, Samuel Groeschel, Wibke G. Janzarik

**Affiliations:** ^1^ Department of Neuropediatrics and Muscle Disorders, Medical Center, Faculty of Medicine University of Freiburg Freiburg Germany; ^2^ Institute of Medical Biometry and Statistics, Faculty of Medicine and Medical Center University of Freiburg Freiburg Germany; ^3^ German PCH Patient Network (PCH‐Familie e.V.) Böblingen Germany; ^4^ Hertie Institute for Clinical Brain Research, Medical Faculty University of Tübingen Tübingen Germany; ^5^ Zoological Institute Karlsruhe Institute of Technology Karlsruhe Germany; ^6^ Institute of Biological and Chemical Systems‐Functional Molecular Systems Karlsruhe Institute of Technology Karlsruhe Germany; ^7^ Heidelberg Academy of Sciences and Humanities Heidelberg Germany; ^8^ Department of Child Neurology and Developmental Medicine University Children's Hospital Tübingen Germany

## Abstract

**Aim:**

To calculate growth charts for pontocerebellar hypoplasia (PCH) type 2A (PCH2A) and compare them to German reference charts, especially with regard to nutritional aspects.

**Method:**

Data were gathered from a cohort of patients with genetically confirmed PCH2A, who were predominantly recruited through the German PCH patient network (65 patients [33 female, 32 male] at a mean age of 8 years 7 months). We collected data retrospectively using a parent questionnaire, and from medical records (December 2020–September 2022). Disease‐specific growth charts were prepared from predominantly longitudinal data using the gamlss package implemented in R. Sex‐disaggregated growth charts for PCH2A were compared to German reference data from the Kinder‐ und Jugendgesundheitssurvey (German Health Interview and Examination Survey for Children and Adolescents).

**Results:**

The height and weight of participants with PCH2A were within the reference range at birth. Mean height was significantly lower at 6 months of age, weight at 3 months, and body mass index at 4 months. These deviations were also mostly significant later on. Head circumference in individuals with PCH2A was significantly below average at birth; all participants showed severe and progressive microcephaly in the further course. Caloric intake was within or above reference values.

**Interpretation:**

Participants with PCH2A exhibited progressive microcephaly and frequently failed to thrive. Disease‐specific growth charts are a helpful tool to monitor those with PCH2A.

## Graphical abstract

To our knowledge, we are the first to provide constructed disease‐specific growth charts for PCH2A (age 0–18 years). Constructed growth charts (0–2 years) for height, weight, body mass index, and head circumference are compared in a sex‐disaggregated manner to typically developing children. Failure to thrive is evident despite normal caloric intake and PEG‐placement in most patients with PCH2A.

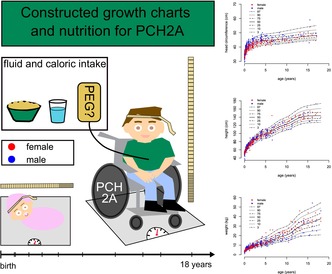

Plain language summary: https://onlinelibrary.wiley.com/doi/10.1111/dmcn.16445

**Abbreviations**
KiGGS: Kinder‐ und Jugendgesundheitssurvey (German Health Interview and Examination Survey for Children and Adolescents)
PCH: pontocerebellar hypoplasia
PCH2A: pontocerebellar hypoplasia type 2A
PEG: percutaneous endoscopic gastrostomy

**What this paper adds**

- Patients with pontocerebellar hypoplasia type 2A (PCH2A) exhibited progressive microcephaly and frequently failed to thrive.
- Reduced thriving in individuals with PCH2A was evident despite normal caloric intake and percutaneous endoscopic gastrostomy tube placement.
- Disease‐specific growth charts are a helpful tool to monitor patients with PCH2A.

Pontocerebellar hypoplasia (PCH) is a diverse group of very rare, heterogeneous disorders.^1^ Among its subtypes, pontocerebellar hypoplasia type 2A (PCH2A)—although rare, with an incidence of less than 1 per 200 000—is the most common.^2^, ^3^ PCH2A, in contrast, represents a genetically homogeneous group of individuals, defined by a specific homozygous pathogenic variant in *TSEN54* (c.919G>T [p.Ala307Ser]), most probably because of a founder effect; it is inherited in an autosomal recessive manner.^2^ Magnetic resonance imaging of individuals with PCH2A typically shows marked hypoplasia of the pons and cerebellum, with a distinctive ‘dragonfly’ appearance, and progressive neocortical atrophy.^4^, ^5^ This finding is further supported by a PCH2A organoid model, which demonstrates the involvement of both cerebellar and neocortical structures.^6^ Individuals with PCH2A typically show profound neurodevelopmental impairment and a characteristic movement disorder with dyskinesia or chorea, which is often accompanied by dystonic attacks or spasticity.^3^ During the further course of the disease, epileptic seizures often occur and can be difficult to treat.^3^ Progressive microcephaly has been reported as a major clinical characteristic of PCH2A, but height and weight gain are also reduced.^3^ In many individuals with PCH2A, conspicuous movements and feeding difficulties are already observed during the neonatal period.^3^ Because of dysphagia, individuals with PCH2A often require percutaneous endoscopic gastrostomy (PEG) to be able to feed.^3^ Feeding problems and impaired growth are a common finding in severe neurodevelopmental disorders.^7^ Disease‐specific growth charts help to monitor the growth of individuals with PCH2A compared with other individuals in the same age group.^8^ For several genetic syndromes (Down syndrome, Turner syndrome, mucopolysaccharidosis type III [Sanfilippo syndrome]) disease‐specific growth charts have already been published.^9^, ^10^, ^11^ However, to date and to the best of our knowledge, no PCH2A‐specific growth charts are available. This study aimed to provide PCH2A‐specific growth charts for height, weight, body mass index (BMI), and head circumference, and compare them to the German Health Interview and Examination Survey for Children and Adolescents (Kinder‐ und Jugendgesundheitssurvey [KiGGS]) reference centiles.^12^ As a follow‐up, we compared survival between females and males. Data on fluid and caloric intake, such as PEG tube placement, were added to identify possible causes of failure to thrive.

## METHOD

### Study design and ethical approval

Data were gathered from a cohort of patients with genetically confirmed PCH2A, predominantly recruited through the German PCH patient network (PCH‐Familie e.V.). Data from the first natural history study were included.^3^ In all cases, written informed consent was given by the legal guardians. We collected data retrospectively using a parent questionnaire, and from medical records (December 2020–September 2022). A final telephone interview with the parents clarified any discrepancies. Anthropometric data were collected from measurements recorded during medical check‐ups at several points in the individual's clinical course and were supplemented by data extracted from the medical records, all performed by health professionals. Additionally, parents completed measurements from their own records. Data on the instruments used were not recorded. Our questionnaire included a protocol for daily caloric intake. Caloric intake was either calculated by parents or estimated based on a detailed account of daily food consumption using product‐specific caloric values. No specific software was used. The study was approved by the ethics committee of the University of Freiburg in November 2020 (no. 20–1040) and by the ethics committee of the University of Tübingen in 2012 (no. 105/2012BO2) and 2021 (no. 961/2020BO2). The study is registered in the Deutsches Register Klinischer Studien (ID: DRKS00022511).

### Statistical analysis

SPSS v29.0.0.0 (IBM Corp., Armonk, NY, USA) and R (R Foundation for Statistical Computing, Vienna, Austria) were used for the statistical analysis. Descriptive statistics (means, medians, SDs, and percentages, as appropriate) were calculated for the demographic and nutritional variables. Growth charts for PCH2A were developed from birth to age 18 years, with outliers included in the analysis. For individuals born preterm, birth measurements were excluded from the analysis and corrected age was used for all further measurements. Three participants (all male) were born preterm. Height, weight, BMI, and head circumference were compared in a sex‐disaggregated manner to anthropometric measurements of typically developing children from a German reference population from the KiGGS.^12^ Because of more extensive data in the first 2 years of life, graphical and statistical comparisons to the reference group were restricted to this age group except for BMI. For BMI, graphical comparison could be made from 4 months to 2 years, because of reference data missing before the age of 4 months, while statistical comparison was made up to the age of 5 years. For comparison, a one‐sample Student's *t*‐test or a Wilcoxon rank‐sum test were used, depending on whether the assumption of a normal distribution was fulfilled or not. Differences in survival were assessed using Kaplan–Meier estimates and a log‐rank test, assuming uninformative censoring. *p <* 0.05 was considered statistically significant. No correction for multiple comparisons was performed.

To calculate the growth charts, lambda‐mu‐sigma models were used.^13^ Specifically, the lambda‐mu‐sigma method in the R package gamlss v5.4–10 was applied.^14^, ^15^, ^16^ For each body measurement, three slightly different models, all based on modifications of the Box–Cox transformation, were fitted to the data. Growth charts were reviewed for medical plausibility to evaluate the model fit, and worm plots and *q*‐statistics were performed.^17^ If no model fit was applicable, a normal model was used instead. Generally, the fitted centiles using various models produced very similar results, indicating the robustness of our approach. For more details on the calculation of the growth charts, see Appendix S1.

### Related publications

A preprint of this article was published on *medRxiv*.^18^ The graphics are displayed in a parent and caregiver guideline of the German PCH patient network.^19^

## RESULTS

### Study population

The data of 65 participants with genetically proven PCH2A were analysed (Table 1), including 33 patients from the first natural history study.^3^ The updated follow‐up for 21 of these participants could be collected, with survival data for five more. No follow‐up information was available for seven participants. Fifty‐six participants originated from Germany; nine came from Switzerland, Austria, and Bulgaria. Nine sibling pairs were included. Sex was equally distributed. Seven of 33 females, and 7 of 32 males, reached survival for up to 15 years. No statistically significant difference in survival was found between the sexes (*p =* 0.4). Right censoring was required for 27 females and 22 males. Overall survival data are presented in Figure S1.

**TABLE 1:** Study population.

	Number of patients (%)	Mean age at the end of the follow‐up (SD), years:months
Total cohort	65/65 (100)	8:7 (7:5)
Sex		
Female	33/65 (50.8)	8:1 (7:2)
Male	32/65 (49.2)	9:2 (7:6)
Survival		
Alive	42/65 (64.6)	8:7 (7:5)
Deceased	16/65 (24.6)	9:0 (8:6)
Unknown	7/65 (10.8)	7:9 (5:11)

### Height

**FIGURE 1:**
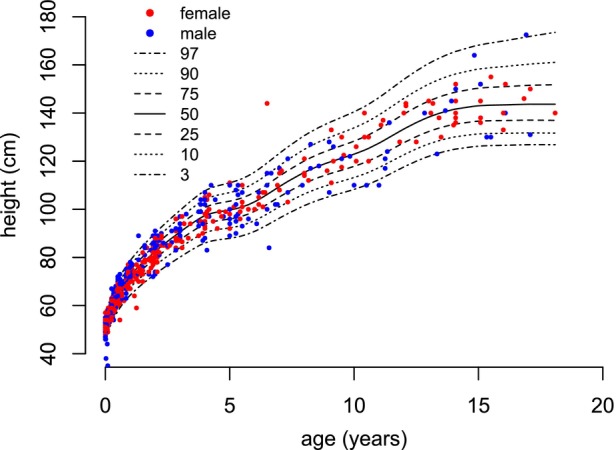
Constructed pontocerebellar hypoplasia type 2A‐specific growth charts for height (0–18 years).

**FIGURE 2:**
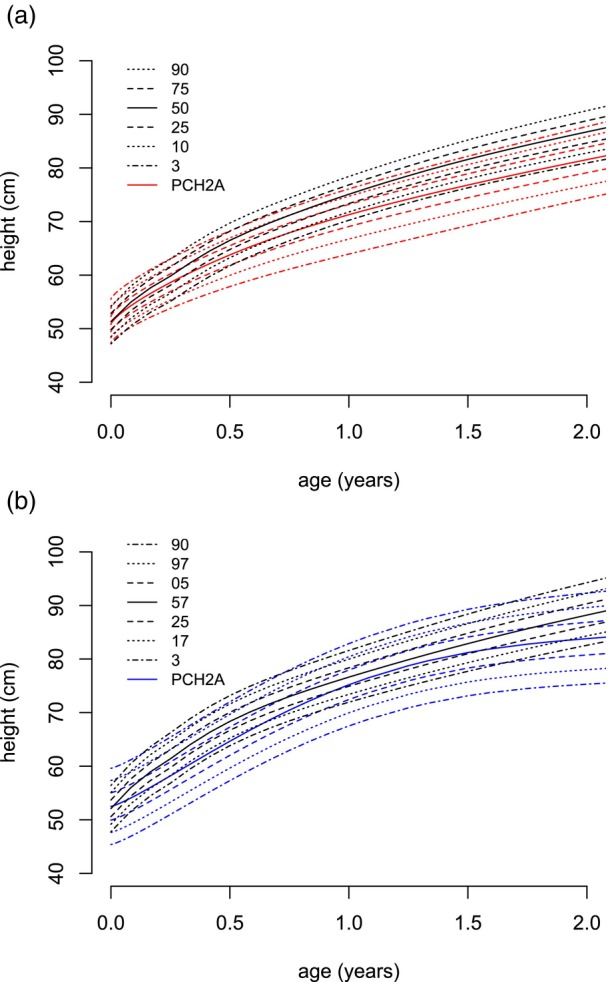
Constructed sex‐disaggregated growth charts for height versus the reference population (black, KiGGS^12^) from 0 to 2 years. (a) Females (red, 233 measurements). (b) Males (blue, 193 measurements). Abbreviations: KiGGS, Kinder‐ und Jugendgesundheitssurvey. PCH2A, pontocerebellar hypoplasia type 2A.

For height, 643 measurements were collected from age 0 to 18 years, that is, 353 measurements from females and 290 from males. The mean number of available measurements per patient was 10.0 (SD = 5.0). For the age span from 0 to 2 years, 426 measurements were available, while from age 2 to 18 years, 217 measurements were available. Between age 15 years and 18 years, 11 measurements for females and six measurements for males were included. For participants with PCH2A aged 0 to 18 years, height centiles were developed without distinguishing between the sexes (Figure 1). For participants with PCH2A aged 0 to 2 years, sex‐disaggregated centiles are displayed in comparison to the reference group (Figure 2). At birth, mean height was 51.4 cm (SD = 2.2 cm) in females and 52.0 cm (SD = 2.3 cm) for males. Both values did not differ significantly from the reference group. With increasing age, height measurements showed a progressive deviation from the reference values, which reached statistical significance at 4 months of age in females (*p* = 0.021), and at 6 months of age in males (*p* = 0.046). In females, height remained below the reference population, with statistical significance from 6 months onwards (*p* = 0.002); in males, this occurred from 12 months onwards (*p* = 0.004). For the exact *p*‐values, see Table S1 (height).

### Weight

**FIGURE 3:**
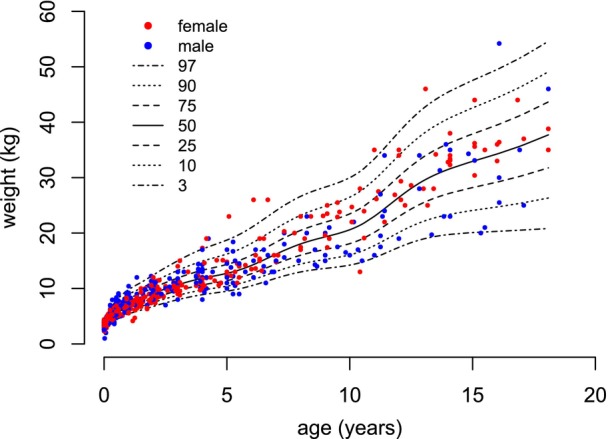
Constructed pontocerebellar hypoplasia type 2A‐specific growth charts for weight (0–18 years).

**FIGURE 4:**
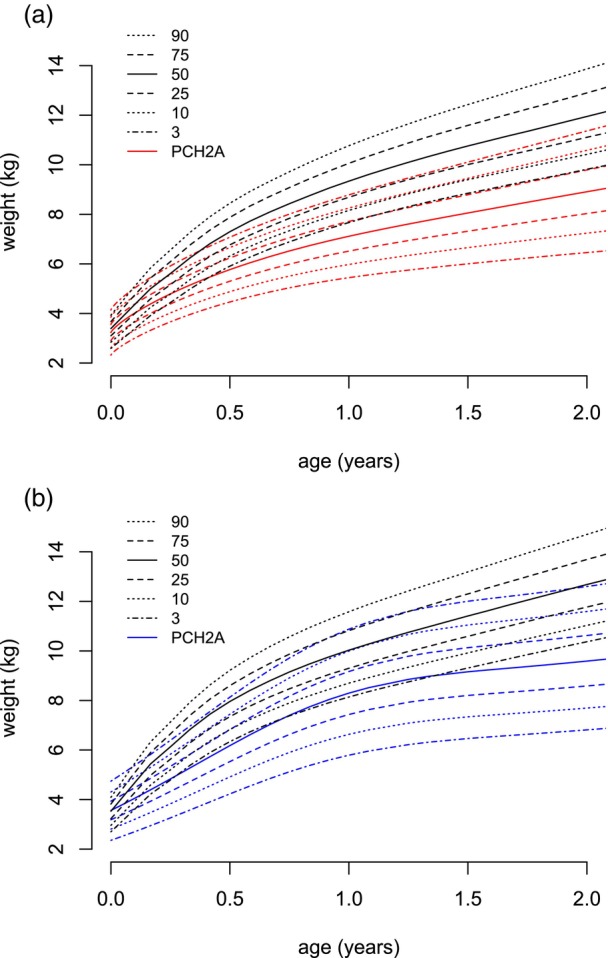
Constructed sex‐disaggregated growth charts for weight versus the reference population (black, KiGGS^12^) from 0 to 2 years. (a) Females (red, 246 measurements). (b) Males (blue, 206 measurements). Abbreviations: KiGGS, Kinder‐ und Jugendgesundheitssurvey; PCH2A, pontocerebellar hypoplasia type 2A.

For weight, 708 measurements were collected from age 0 to 18 years, that is, 385 measurements from females and 323 from males. The mean number of available measurements per participant was 10.9 (SD = 5.1). From age 0 to 2 years, 452 measurements were available; from age 2 to 18 years, 256 measurements were available. In the group aged 15 to 18 years, 12 measurements for females and nine measurements for males were included. For participants with PCH2A aged 0 to 18 years of age, weight centiles were developed without sex disaggregation (Figure 3). For patients with PCH2A aged 0 to 2 years, sex‐disaggregated centiles are displayed in comparison to the reference group (Figure 4). At birth, mean weight was 3.4 kg (SD = 0.4 kg) in females and 3.5 kg (SD = 0.4 kg) in males; both values did not differ significantly from the reference group. Weight measurements progressively diverged from the reference values over time. The difference to the reference values reached statistical significance at age 3 months in females (*p* = 0.004) and at age 1 month in males (*p* = 0.038), and remained statistically significant. For the exact *p*‐values, please refer to Table S1 (weight).

### Body mass index

**FIGURE 5:**
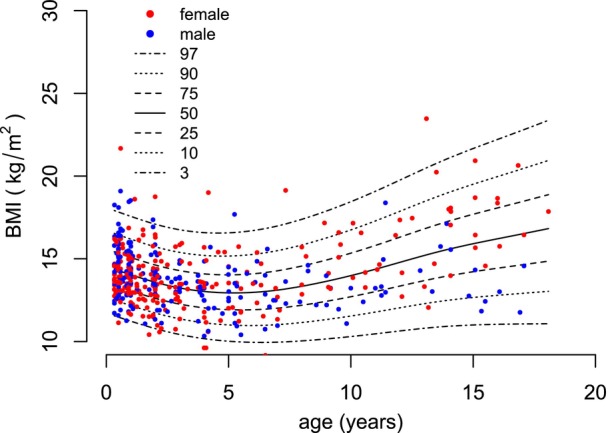
Constructed pontocerebellar hypoplasia type 2A (PCH2A)‐specific growth charts for body mass index (BMI) (4 months–18 years).

**FIGURE 6:**
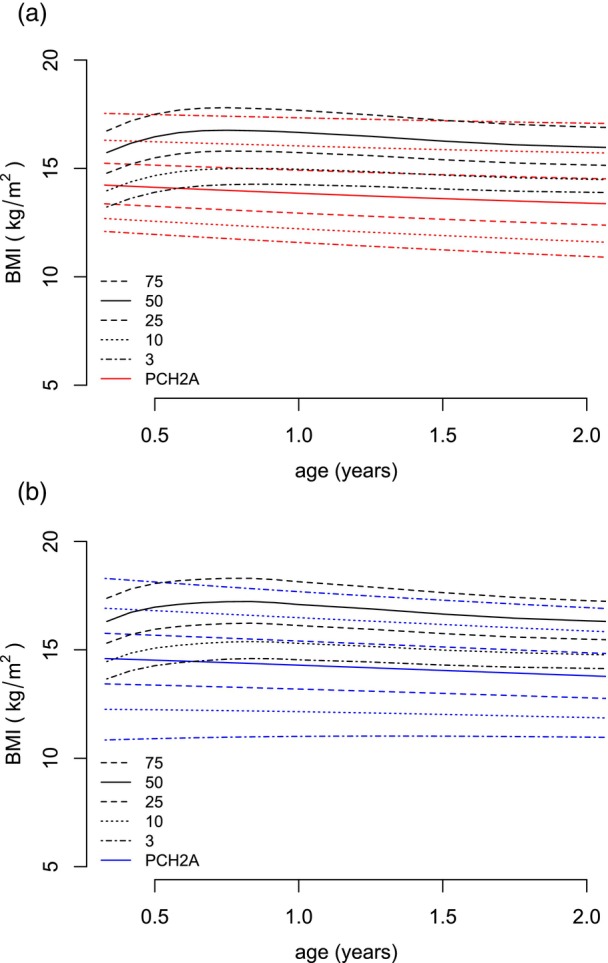
Constructed sex‐disaggregated growth charts for body mass index (BMI) versus the reference population (black, KiGGS^12^) from 4 months to 2 years. (a) Females (red, 128 measurements). (b) Males (blue, 96 measurements). Abbreviations: KiGGS, Kinder‐ und Jugendgesundheitssurvey (German Health Interview and Examination Survey for Children and Adolescents); PCH2A, pontocerebellar hypoplasia type 2A.

For BMI, 440 measurements were calculated from age 4 months to 18 years, that is, 248 values for females and 192 for males. The mean number of available measurements per patient was 7.0 (SD = 4.3). From age 4 months to 2 years, 224 measurements were available; from age 2 to 18 years, 216 measurements were available. Between the ages of 15 and 18 years, 11 values for females and six values for males were included. For participants with PCH2A aged from 4 months to 18 years, BMI centiles were developed without sex disaggregation (Figure 5). For patients with PCH2A aged from 4 months to 2 years, sex‐disaggregated centiles in comparison to the reference group are provided (Figure 6). At 4 months of age, the mean BMI was 14.1 kg/m^2^ (SD = 1.1 kg/m^2^) in females and 14.3 kg/m^2^ (SD = 2.0 kg/m^2^) in males. The BMI of participants with PCH2A showed early deviation from the reference data, with statistical significance already achieved at the first data point, that is, at 4 months of age, in both females and males with PCH2A (females: *p* < 0.001, males: *p* = 0.012). BMI values remained below the reference, with statistical significance from 24 months onwards in females (*p* = 0.048) and from 6 months onwards in males (*p* < 0.001). For the exact *p*‐values, please refer to Table S1 (BMI).

### Head circumference

**FIGURE 7:**
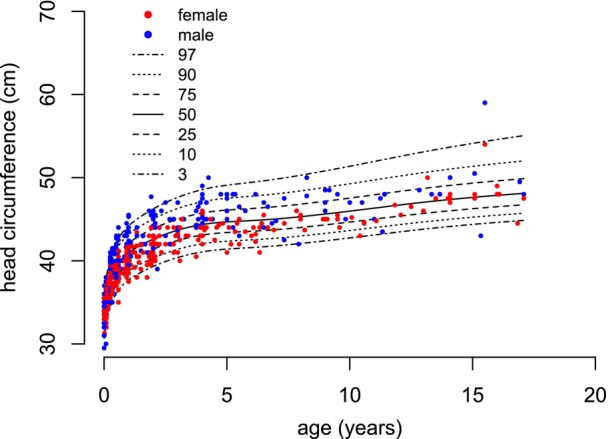
Constructed pontocerebellar hypoplasia type 2A‐specific growth charts for head circumference (0–18 years).

**FIGURE 8:**
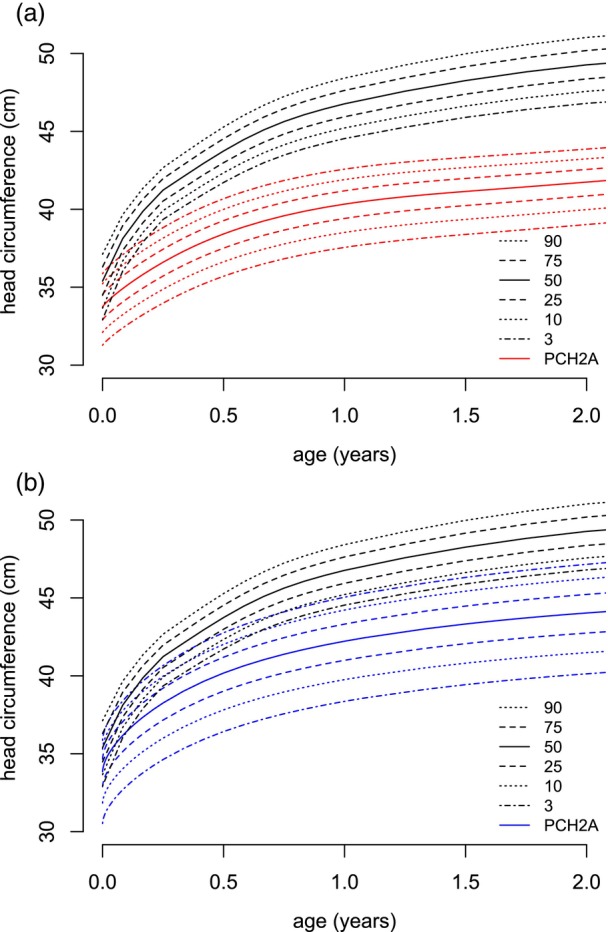
Constructed sex‐disaggregated growth charts for head circumference versus the reference population (black, KiGGS^12^) from 0 to 2 years. (a) Females (red, 224 measurements). (b) Males (blue, 196 measurements). Abbreviations: KiGGS, Kinder‐ und Jugendgesundheitssurvey; PCH2A, pontocerebellar hypoplasia type 2A.

Six hundred measurements for head circumference were collected from age 0 to 18 years, that is, 320 values for females and 280 for males. The mean number of available measurements per participant was 9.5 (SD = 4.4). From age 0 to 2 years, 420 measurements were available; from age 2 to 18 years, 180 measurements were available. Between 15 years of age and 18 years of age, eight measurements for females and six measurements for males were included. The centiles of head circumference for participants with PCH2A up to 18 years were developed (Figure 7). For participants with PCH2A aged 0 to 2 years, sex‐disaggregated centiles in comparison to the reference group are provided (Figure 8). At birth, the mean head circumference of females and males with PCH2A was 33.8 cm (SD = 1.2 cm) and 34.1 cm (SD = 1.1 cm) respectively. Both values differed significantly from the reference group already at birth (both *p* < 0.001) and remained below the reference values with increasing age. Rapid head circumference growth was evident during the initial years of life; head circumference increased across all centiles throughout childhood. Males with PCH2A exhibited a higher head circumference and higher variability of head circumference than females at the same age. For the exact *p*‐values, please refer to Table S1 (head circumference).

### Feeding and caloric intake

**FIGURE 9:**
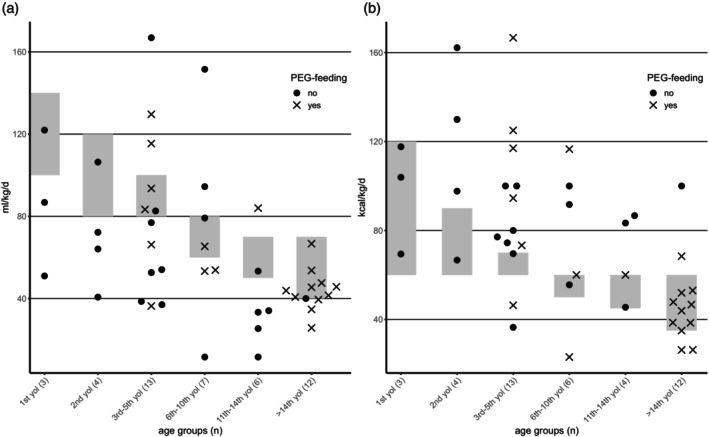
(a) Individual fluid intake in ml/kg/d (*n* = 45). (b) Individual caloric intake in kcal/kg/d (*n* = 42). Both were compared to age‐dependent recommendations (grey bars) after Jochum^20^ (year[s] of life; yol). Abbreviation: PEG, percutaneous endoscopic gastrostomy.

Of the 65 participants, 37 received a PEG feeding tube; 26 of these participants were at least partly fed continuously via PEG feeding tube. Feeding via PEG tube showed only limited effect on weight gain in individual participants (Figure S2). Tendency‐wise, participants with early PEG tube feeding, that is, within the first 5 years of life, were in a higher range of weight and BMI (data not shown). Information on fluid intake for 45 participants was available; caloric intake for 42 participants was also available. Daily intake of fluids and calories differed greatly between individual participants with PCH2A (Figure 9). As a reference, the age‐dependent recommendations from Jochum were used.^20^ In many participants, caloric intake was within or even above reference values, whereas fluid intake was often below reference values. Of participants with low fluid intake, most had not yet received PEG tube feeding. Twenty‐six of 29 families stated that they would choose to have a PEG feeding tube inserted again. The main advantages cited included less stressful eating, reduced pressure during meals, and easier medication administration. Additionally, families noted that the tube was life‐sustaining and associated with fewer gastrointestinal issues and (respiratory) infections.

## DISCUSSION

PCH2A is a rare neurodevelopmental disorder characterized by dysphagia, severe gastrointestinal symptoms, and failure to thrive.^21^, ^22^ This study aimed to provide PCH2A‐specific growth charts, and data on nutritional aspects, to support clinical monitoring and care of individuals with PCH2A. Disease‐specific centiles provide a helpful tool for clinicians and affected families; they have already been published for several rare neurodevelopmental diseases using a similar methodological approach.^11^, ^23^

Consistent with published data, the height and weight of females and males with PCH2A were within the normal range at birth, but progressively fell behind as individuals aged.^3^, ^24^ In a previous natural history study, mean height was within the normal range until the age of 4 years, while the height of several individuals with PCH2A was already below the normal range from 1 year of age onwards.^3^ This is in agreement with our data.

In the first years of life, weight gain was more affected than height, with many individuals failing to thrive despite PEG tube placement and sufficient caloric intake. This can be partially attributed to severe feeding problems, gastro‐oesophageal reflux, and vomiting, which are commonly observed in individuals with PCH2A at an early age.^3^ The BMI of individuals with PCH2A initially decreased from birth to age 5 years, before increasing again. From clinical experience, the gastrointestinal symptoms of individuals with PCH2A tend to improve with increasing age (unpublished data). Moreover, individuals with more severe PCH2A, that is, showing more severe gastrointestinal and feeding problems at an early age, might have a higher mortality within the first years of life.

Caloric intake showed great variability, which is primarily explained by clinical differences, such as varying gastrointestinal symptoms. Having milder PCH2A or the addition of calories to compensate for vomiting most probably explain outliers in the upper range. Although the disease is genetically very homogeneous, there is a spectrum of clinical symptoms. Other factors may also modify the manifestation of the disease (e.g. genetic, epigenetic, environmental).

Overall survival did not differ between females and males. When compared, females showed better weight gain and higher BMI than males. This could be because more females in our cohort started early PEG tube feeding; it could also be due to population bias. In contrast, head circumference, which is less dependent on caloric intake, was higher in males than in females, as it is in typically developing children.^25^

As expected from previous descriptions, patients with PCH2A showed severe and progressive microcephaly.^3^, ^4^ The mean head circumference of patients with PCH2A was already below average at birth, although individual measurements of head circumference were often still within the lower normal range, as already reported.^3^, ^4^, ^24^ Head growth is dependent on the growth of brain structures; progressive microcephaly reflects a reduced growth rate of supratentorial structures and probably some degree of neurodegeneration over time.^5^, ^26^

The small sample size in our study limits robust statistical comparisons of participants fed using a PEG tube and those not fed using a PEG tube. Participants with different diets were not explicitly compared, as in Fraser et al.,^27^ who found a higher caloric intake in PEG‐tube‐fed individuals when a home‐blended diet was administered compared to a formula‐fed diet.^27^ Moreover, individuals fed with a home‐blended diet had a lower gastrointestinal symptom burden.^27^ Therefore, a home‐blended diet in PEG tube feeding is recommended if gastrointestinal symptoms are a key concern.^27^

The study has several limitations. Anthropometric measurements were obtained from different health professionals and parents, without documentation of the instruments used or the use of a standardized programme to calculate caloric intake, thus limiting standardization. Because of the extreme rarity of the disease, no better option was available. The study population was small but within the range of other studies of patients with rare diseases.^11^, ^23^ For individual study participants, several different measurements were available. Therefore, individual growth courses affected the centiles to differing extents. Data were collected from a predominantly German cohort, including nine pairs of siblings, and follow‐up data for seven patients was missing. This could have biased the results. Data were compared to a German reference cohort, showing that a failure to thrive is part of PCH2A. However, the presented PCH2A‐specific centiles can provide a reference for other individuals with PCH2A. In our study cohort, most data were collected in the first 2 years of life. Therefore, graphical and statistical comparison with a reference group was restricted to this early developmental age. In the older age group, not enough data were available to provide sex‐disaggregated centiles. Beyond 15 years of age, only few data could be included in our study. Especially in the older age group, PCH2A‐specific growth charts should be supplemented with more solid data in the future. The centiles represent a highly homogeneous group because all patients carry the same homozygous pathogenic variant, thus minimizing genetic bias. As this represents an unselected cohort of individuals, factors such as comorbid diseases or severe failure to thrive in individuals may influence the centiles. On the other hand, failure to thrive is part of the disease, so it is difficult to choose this as an exclusion criterion.

In conclusion, growth and nutritional status are critical concerns in severe neurological diseases such as PCH2A. Families often lack information on how other patients with the same condition are thriving. They face significant pressure to help their child gain weight, despite the challenges in achieving growth comparable to typically developing children, even with adequate caloric intake or PEG tube placement. Although disease‐specific growth charts are not intended as recommendations—reflecting the care reality of a cohort rather than an ideal scenario—they provide valuable guidance for both families and health care practitioners.

## Supporting information


**Appendix S1:** Detailed information on the calculation of the growth charts.

**Figure S1:** Overall survival of total cohort (*n* = 65) with PCH2A.

**Figure S2:** Individual weight development for patients with early start of PEG feeding (*n* = 16).

**Table S1:** Statistical details regarding height, weight, BMI, and head circumference of patients.

## Data Availability

Data available on request due to privacy/ethical restrictions.
