# Supplementary material for: Constructed growth charts and nutrition for pontocerebellar hypoplasia type 2A
Source: Dev Med Child Neurol. 2025 Jul 15;68(1):82–90. doi: 10.1111/dmcn.16394 (PMC12683303; doi:10.1111/dmcn.16394)
Supplement: Supplementary file 1 — Appendix S1: Detailed information on the calculation of the growth charts. [file DMCN-68-82-s001.docx]

## Calculation of growth charts (Appendix S1)

The LMS method allows to fit smooth centile curves to given data. It is based on the assumption that the distribution of measurements changing according to age can be modelled by suitable transformation of the median, coefficient of variation and skewness of the distribution.^1^ The LMS method within the gamlss package is based on Box-Cox transformations, where the lambda (λ) corresponds to the skewness and is expressed as power in the Box-Cox transformation, mu (μ) corresponds to the Median and sigma (σ) to the generalized coefficient of variation.^1–4^  Transformation for the raw measurements available in R include Box-Cox Cole and Green distribution (BCCGo), Box-Cox Power Exponential distribution (BCPEo) and Box-Cox t distribution (BCTo). ^3,5^

All default-parameters of the models, except “trans.x” and “k” were accepted. “Trans.x” describes the power transformation along the x-axis.^4^ Generally, body measurements show a skewed distribution over time.^6^ Setting “trans.x” as “TRUE” instead of “FALSE” (the default) was used to apply a transformation to the x-axis to normalise this skewed distribution.^4^ “K” describes a penalty in the calculation of the generalised Akaike information criterion, which is applied internally in the LMS method to evaluate goodness of fit and model convergence.^3,4^ The parameter was set to three instead of two (the default) according to the recommendation of Stasinopoulos et al., which is based on an automatic hyperparameter optimisation proposed in Rigby and Stasinopoulos.^4,7^ To consider all available data points, a negligible random small value was added as jittering to every age value.^8,9^

To evaluate model fit, for q-statistics, recommended intervals were accepted, while for worm plots, interval-range was set to nine.^10^ We applied the following criteria for fitting the gamlss models. Only models reaching convergence were accepted. If convergence could not be reached in BCCGo, BPEo, and BCTo, “k” was set back to the default (k=2). In some cases, even with “k” set to two, no model convergence was achieved. In these instances, a normal model was used, which fits a normal distribution to the data, considering only mu and sigma. Consequently, it corresponds to a linear regression model.^10^

For construction of PCH2A-specific growth charts for height, weight, BMI, such as sex-disaggregated growth charts for weight and BMI of males, BCTo distribution showed the best fit. For construction of PCH2A-specific growth charts for hc, such as sex-disaggregated growth charts for height and BMI of females and hc of males, BCCGo distribution showed the best fit. For construction of sex-disaggregated growth charts for height of males and weight of females, Normal distribution showed the best fit. For construction of sex-disaggregated growth charts for hc of females, BCTo distribution showed the best fit with k=2.

## References

1.  Cole TJ, Green PJ. Smoothing reference centile curves: the LMS method and penalized likelihood. *Statistics in medicine* 1992; **11**: 1305–19.

2. Cole TJ. Fitting Smoothed Centile Curves to Reference Data. *Journal of the Royal Statistical Society* 1988; **151**: 385–418.

3. Rigby RA, Stasinopoulos DM. Generalized additive models for location, scale and shape. *Applied Statistics* 2005; **54**: 507–54.

4. Stasinopoulos M, Rigby B, Voudouris V, Heller G, de Bastiani F. Chapter 13: Centile Estimation. In: Chambers JM, Hothorn T, Lang DT, Wickham H, editors. Flexible regression and smoothing: Using GAMLSS in R. Boca Raton, London, New York: CRC Press Taylor & Francis Group a Chapman & Hall book, 2017: 449–98.

5. Stasinopoulos M, Rigby B, Voudouris V, Heller G, Bastiani F de. Chapter 2: Introduction to the gamlss packages. In: Chambers JM, Hothorn T, Lang DT, Wickham H, editors. Flexible regression and smoothing: Using GAMLSS in R. Boca Raton, London, New York: CRC Press Taylor & Francis Group a Chapman & Hall book, 2017: 31–56.

6. Cole TJ. The LMS method for constructing normalized growth standards. *European journal of clinical nutrition* 1990; **44**: 45–60.

7. Rigby RA, Stasinopoulos DM. Using the Box-Cox t distribution in GAMLSS to model skewness and kurtosis. *Statistical Modelling* 2006; **6**: 209–29.

8. Chambers JM, Cleveland WS, Kleiner B, Tukey PA. Studying Tow-Dimensional Data, In: Chambers JM, editor. Graphical methods for data analysis. Boca Raton, FL: Chapman and Hall/CRC an imprint of Taylor and Francis 2018: 75-128.

9. Chambers JM, Hastie TJ. Statistical Models in S. New York: Routledge 1992.

10. Stasinopoulos M, Rigby B, Voudouris V, Heller G, Bastiani F de. Chapter 12: Diagnostics. In: Chambers JM, Hothorn T, Lang DT, Wickham H, editors. Flexible regression and smoothing: Using GAMLSS in R. Boca Raton, London, New York: CRC Press Taylor & Francis Group a Chapman & Hall book, 2017: 417–46.
