# Supplementary figures and images for: Constructed growth charts and nutrition for pontocerebellar hypoplasia type 2A
Source: Dev Med Child Neurol. 2025 Jul 15;68(1):82–90. doi: 10.1111/dmcn.16394 (PMC12683303; doi:10.1111/dmcn.16394)

probability of survival

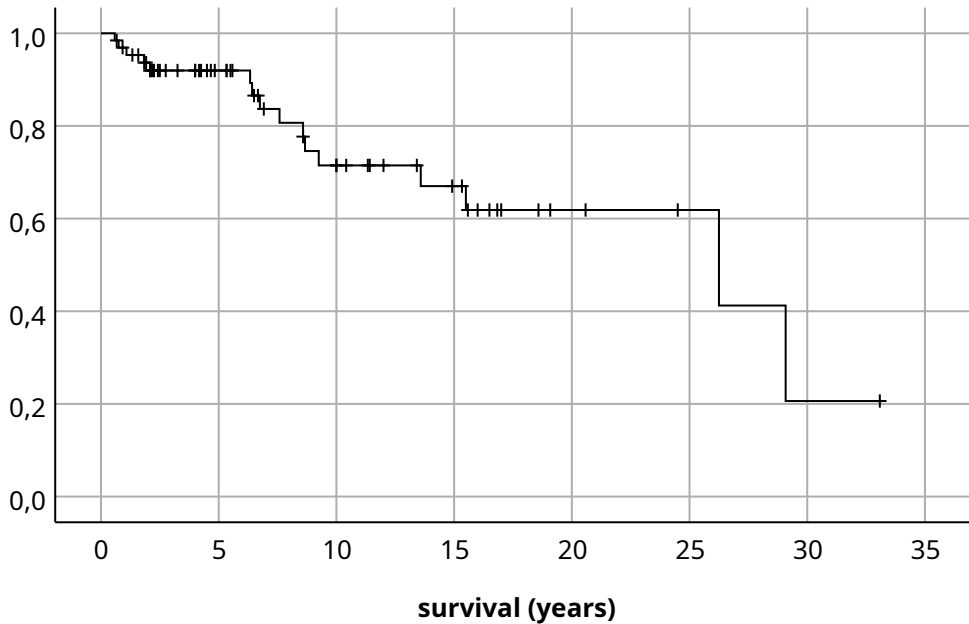

Supplement: Supplementary file 2 — Figure S1: Overall survival of total cohort (n = 65) with PCH2A. [file DMCN-68-82-s002.pdf]

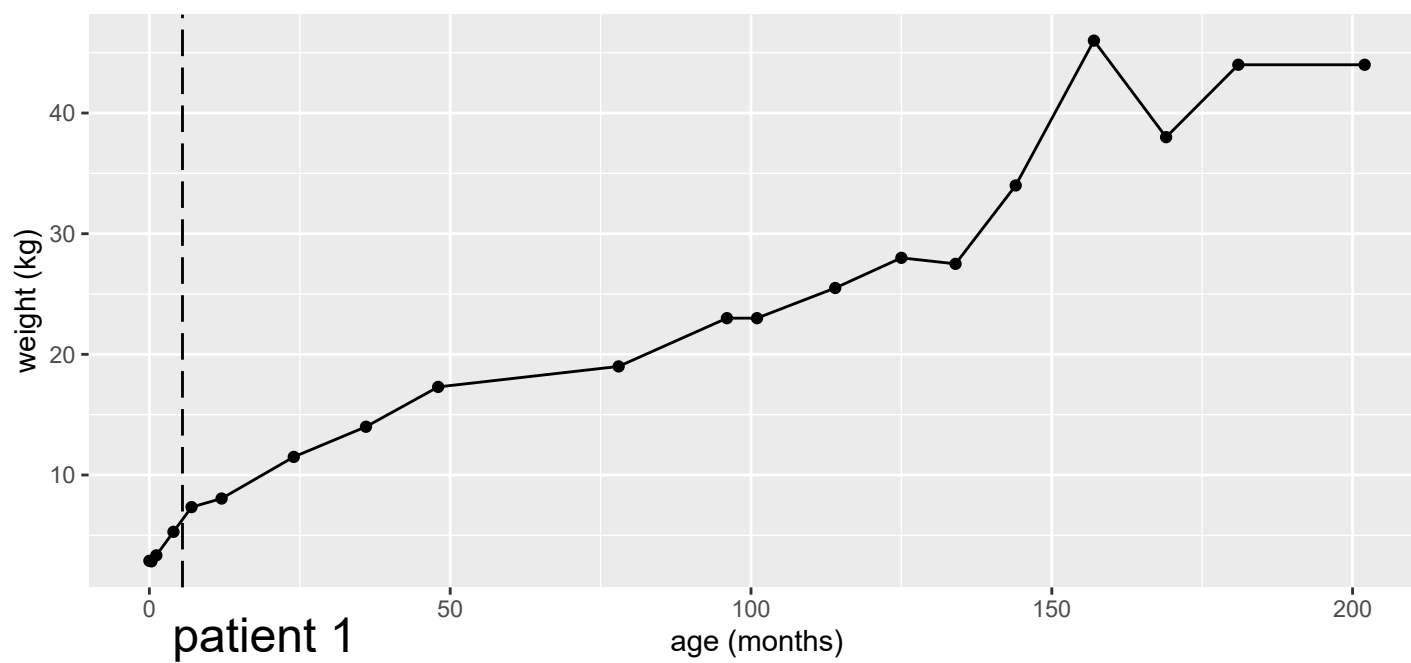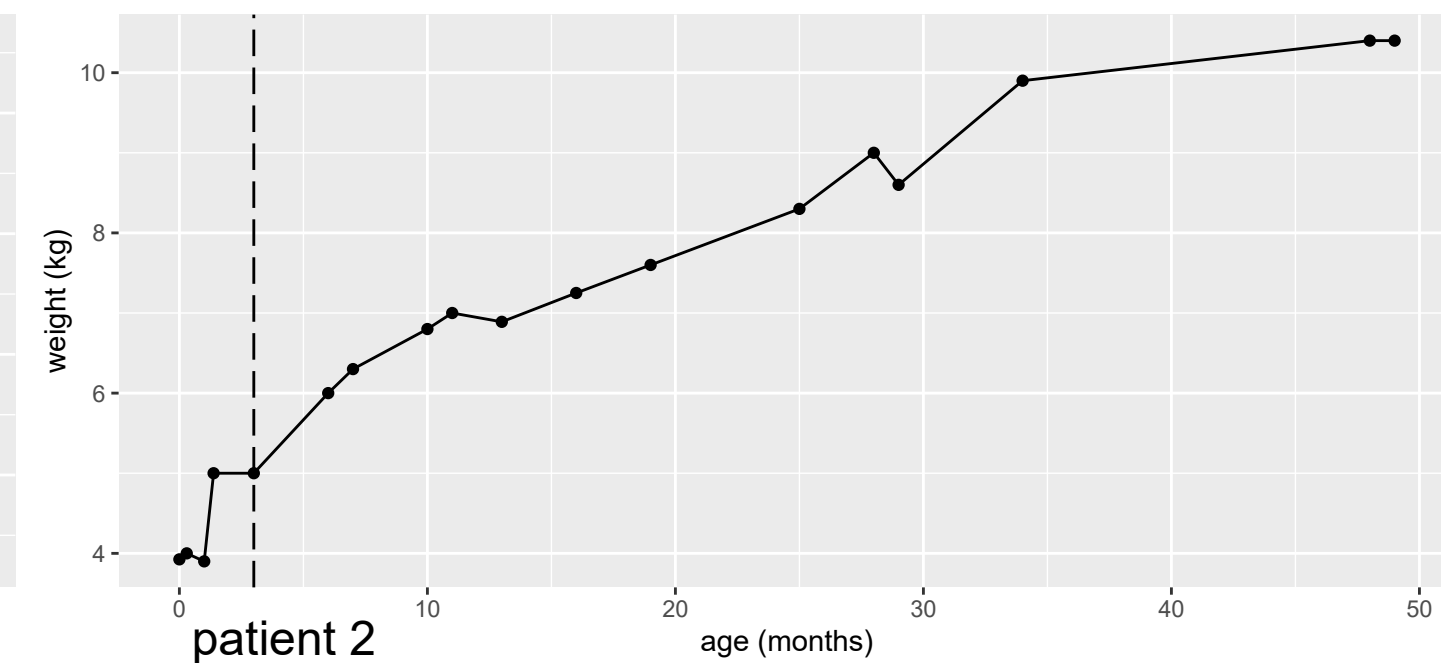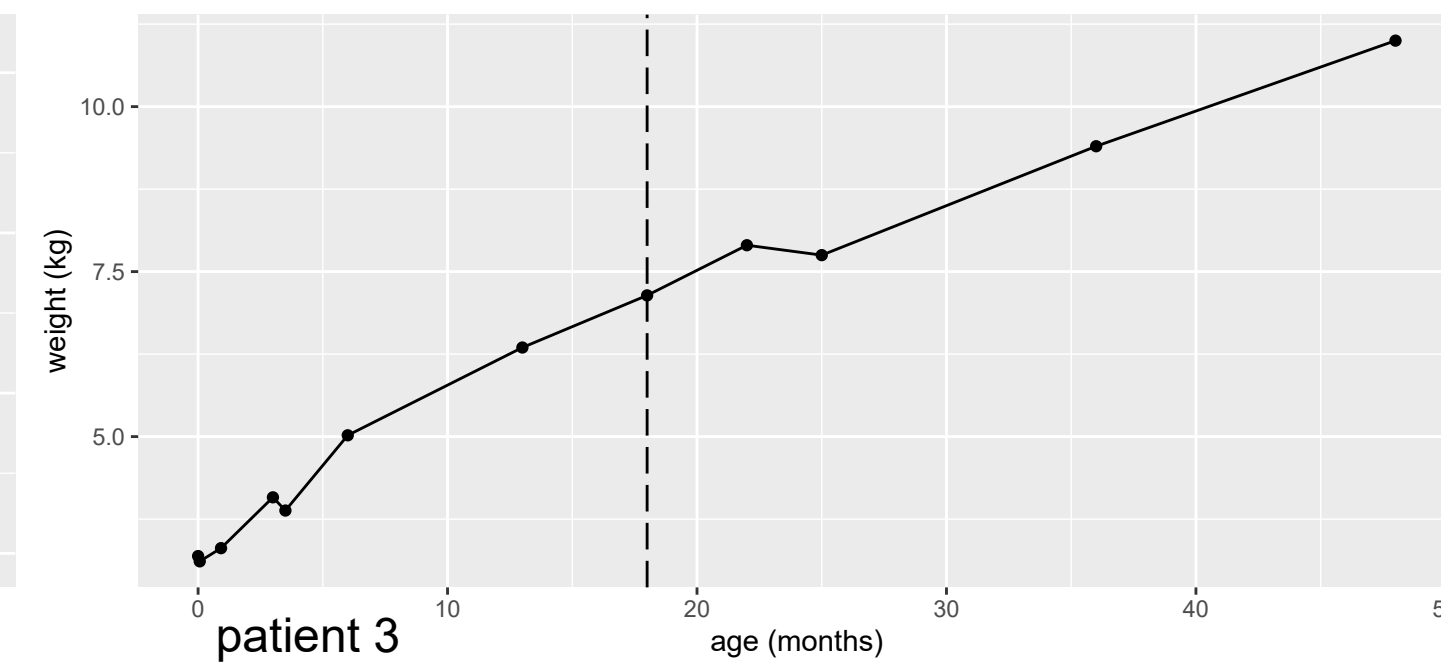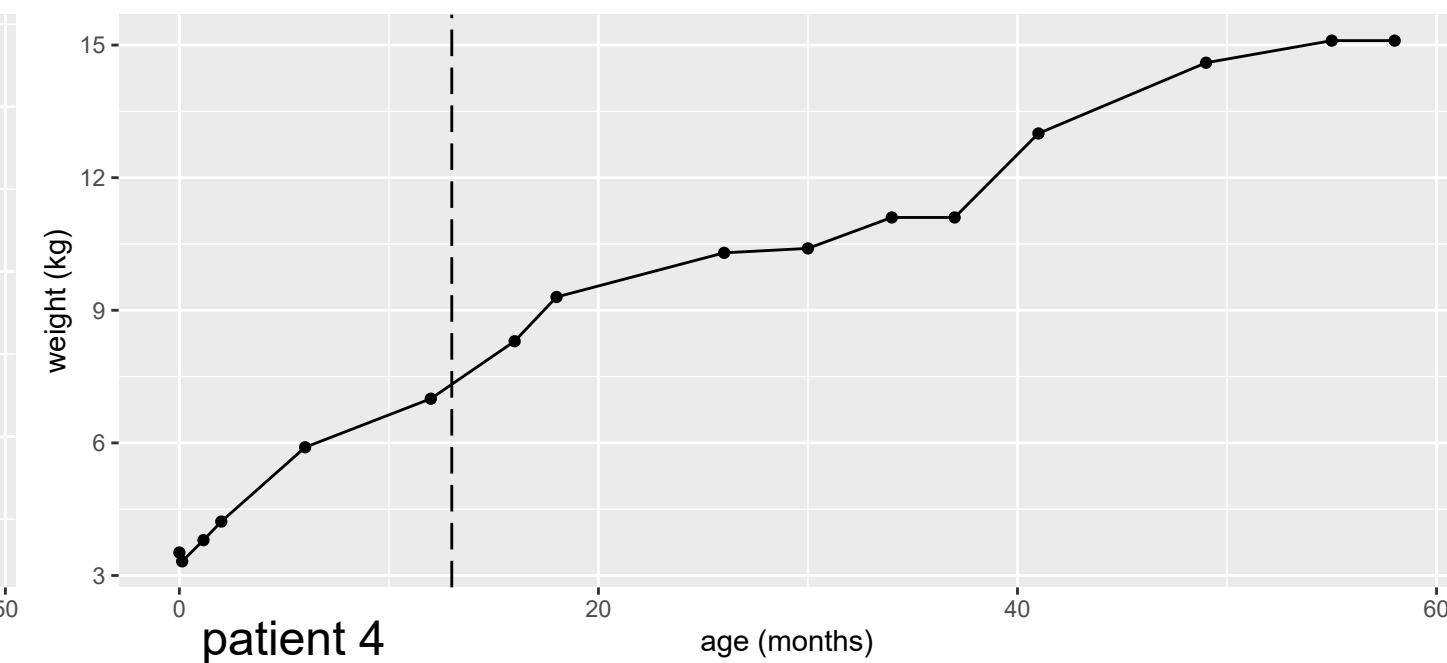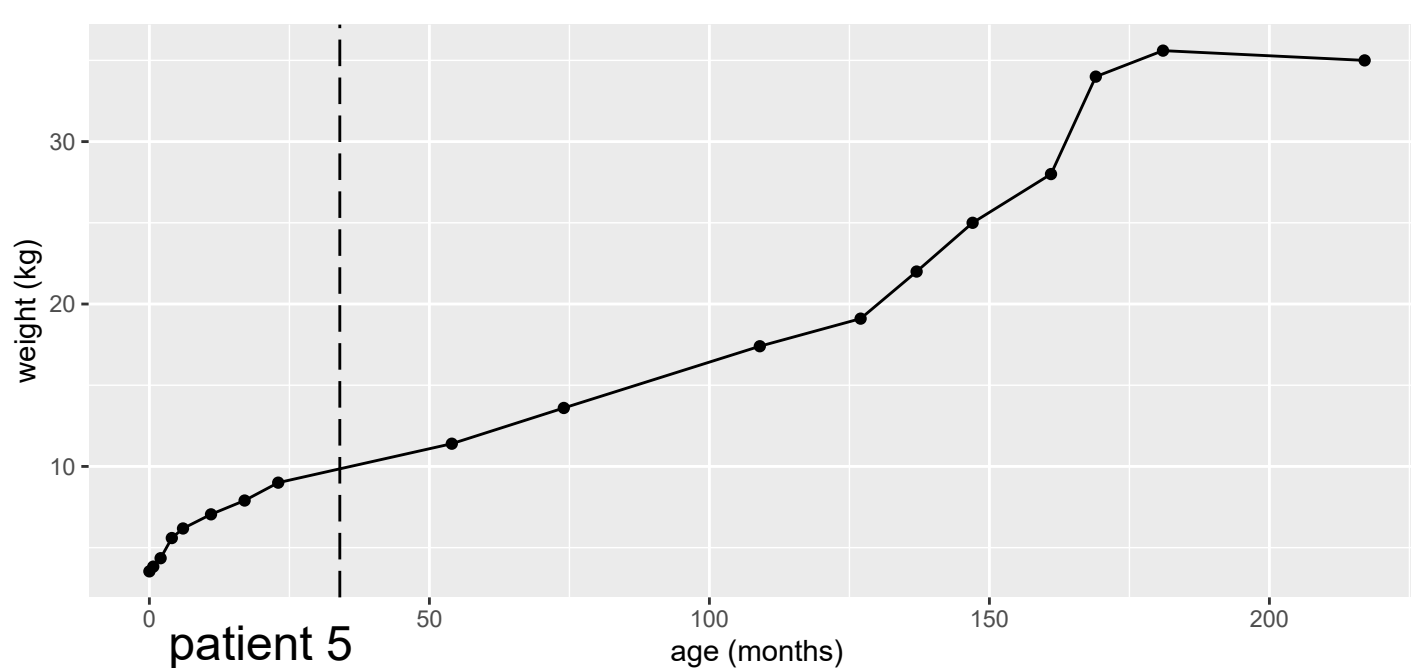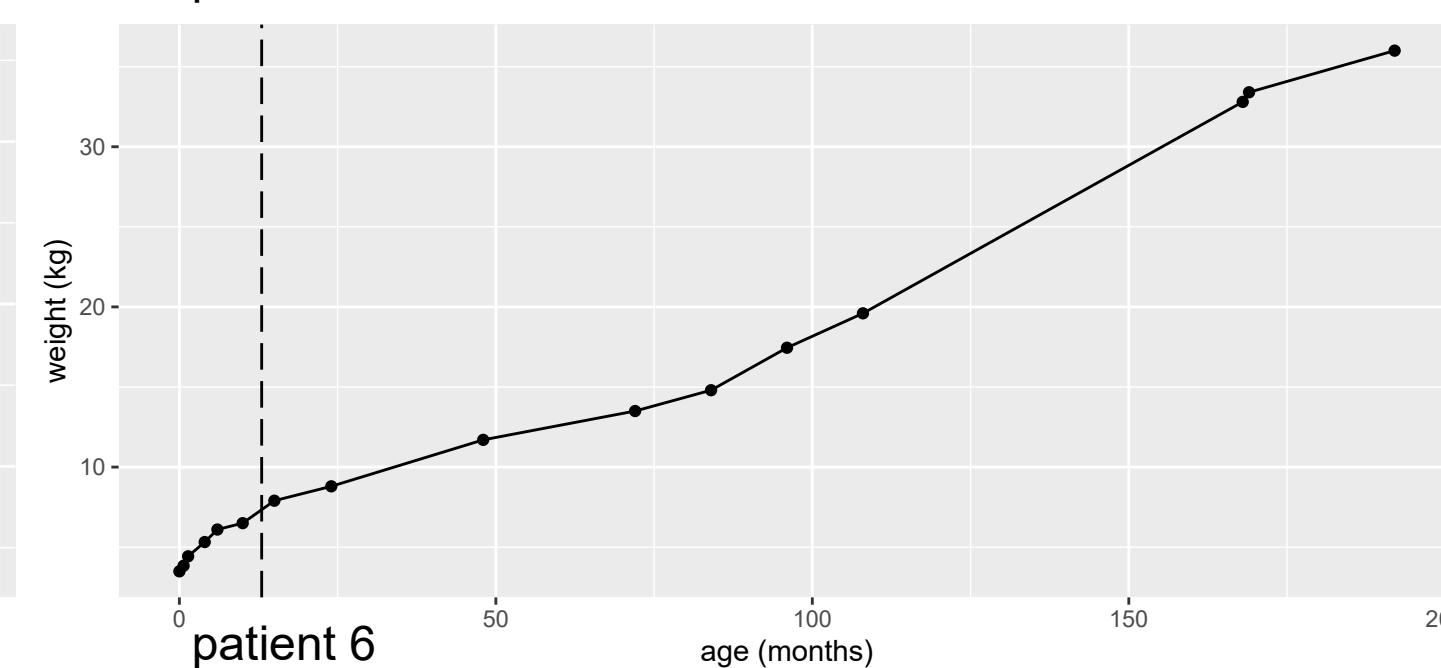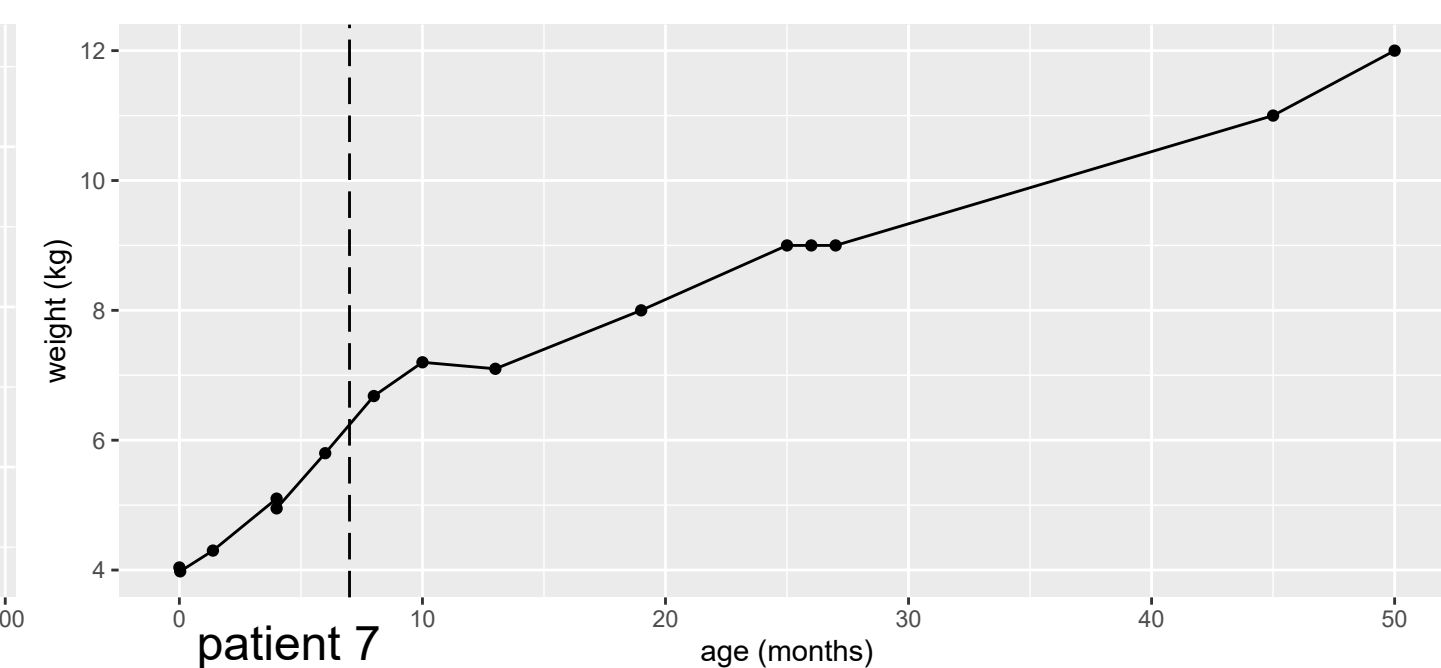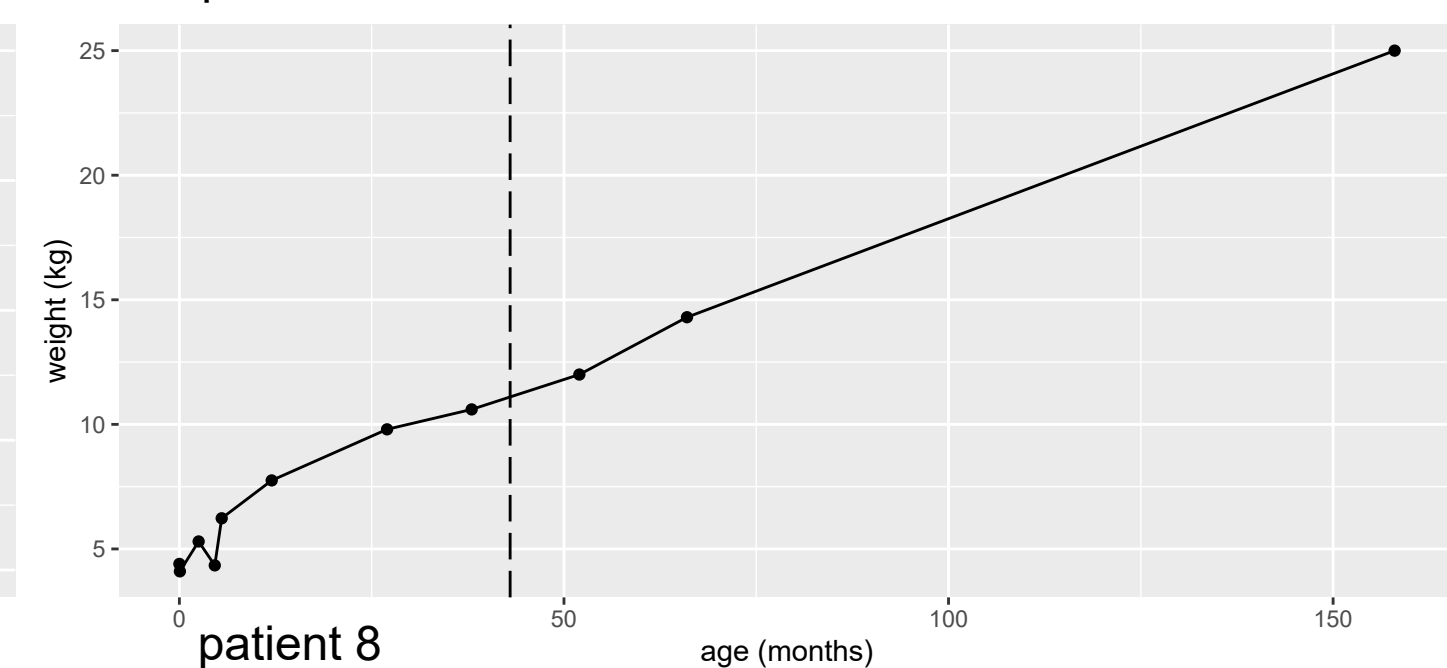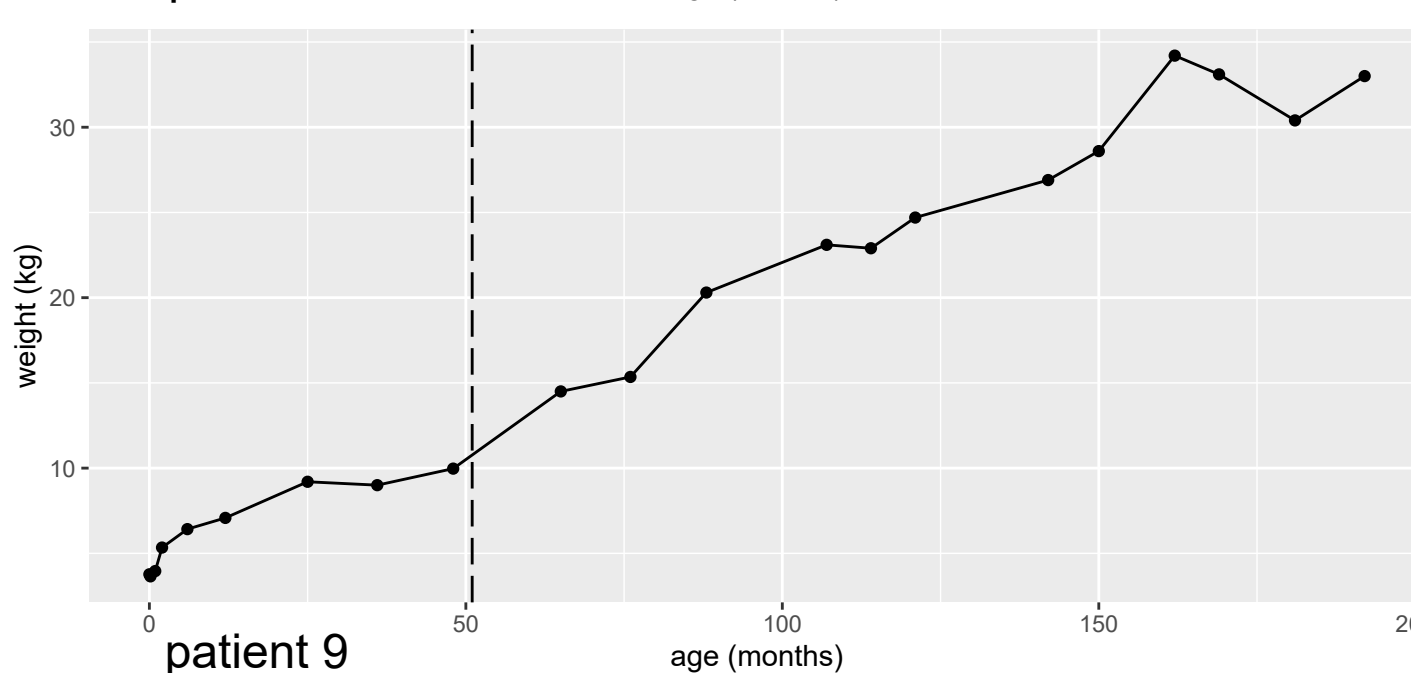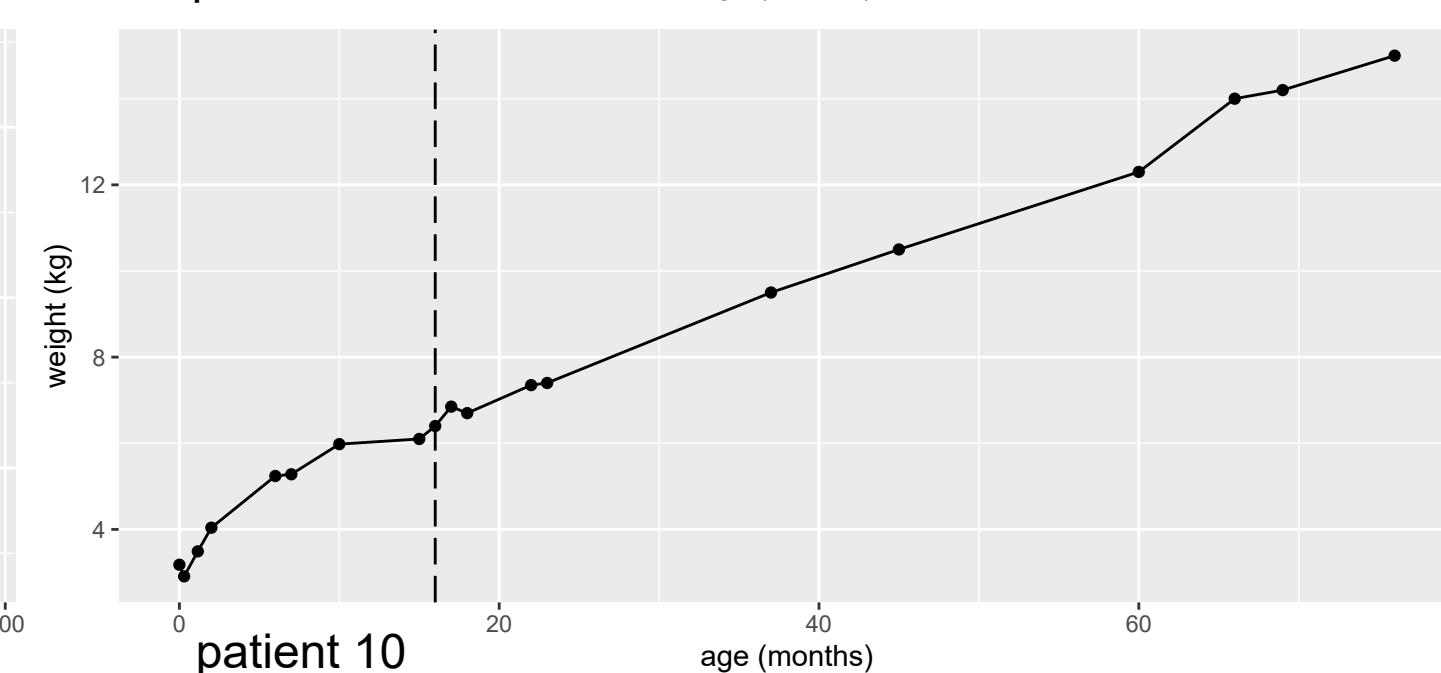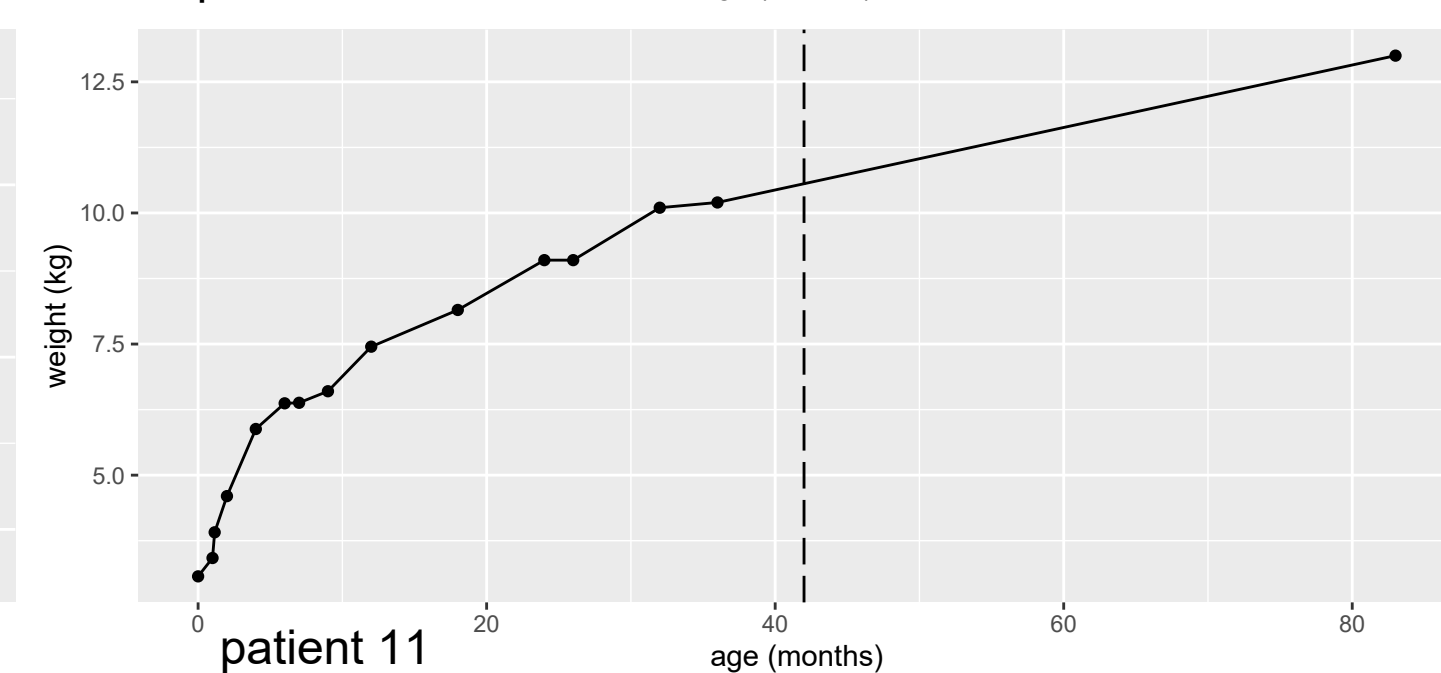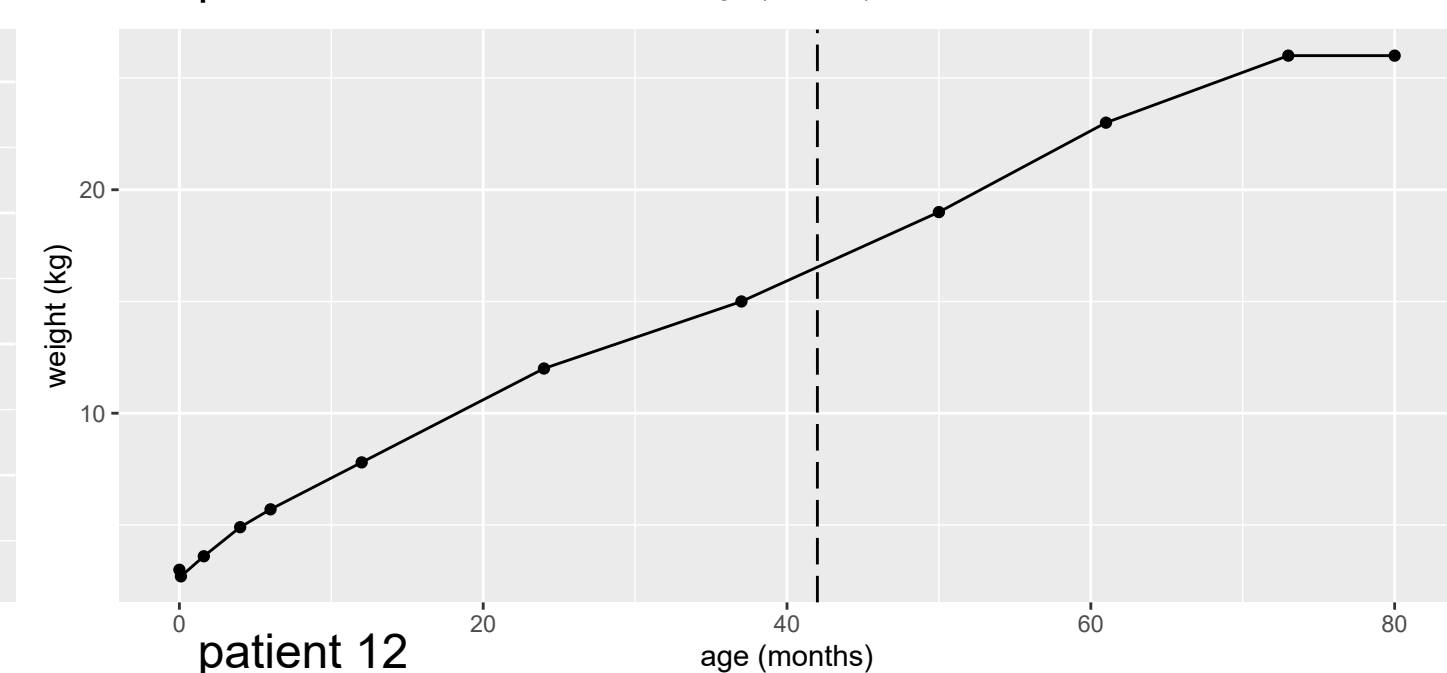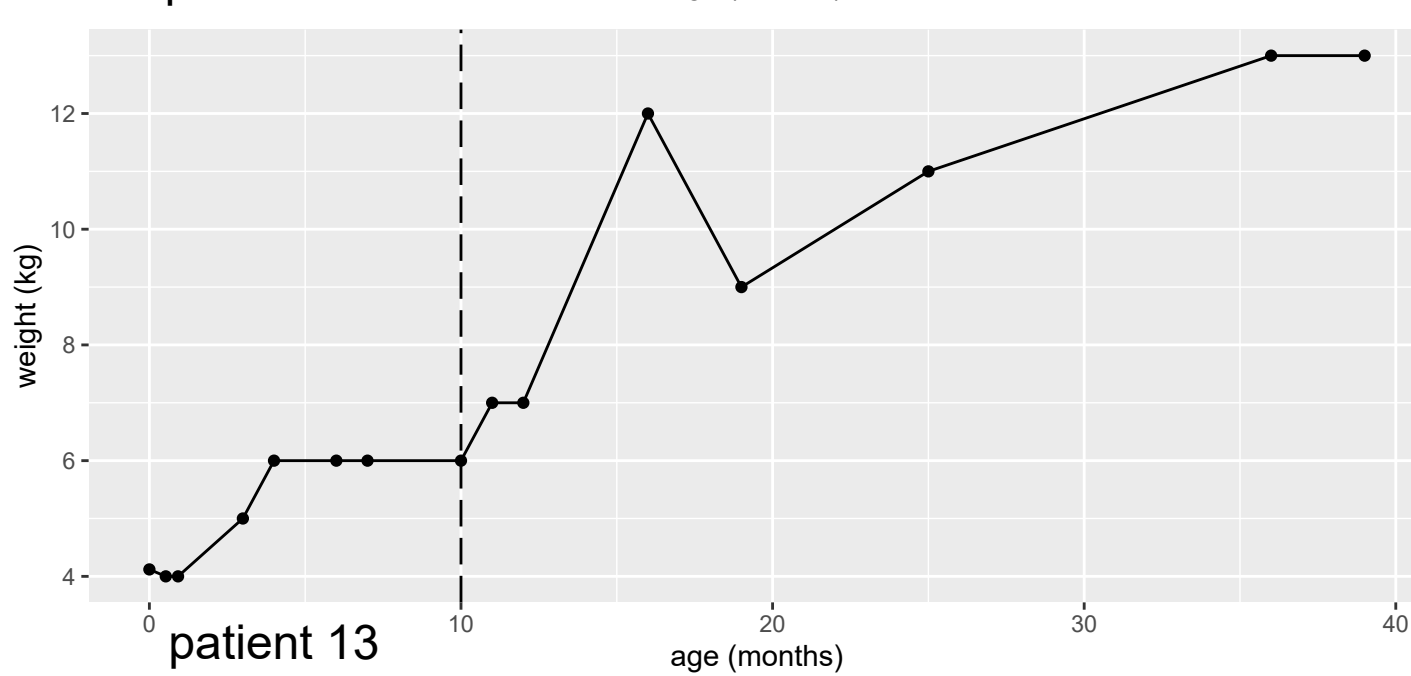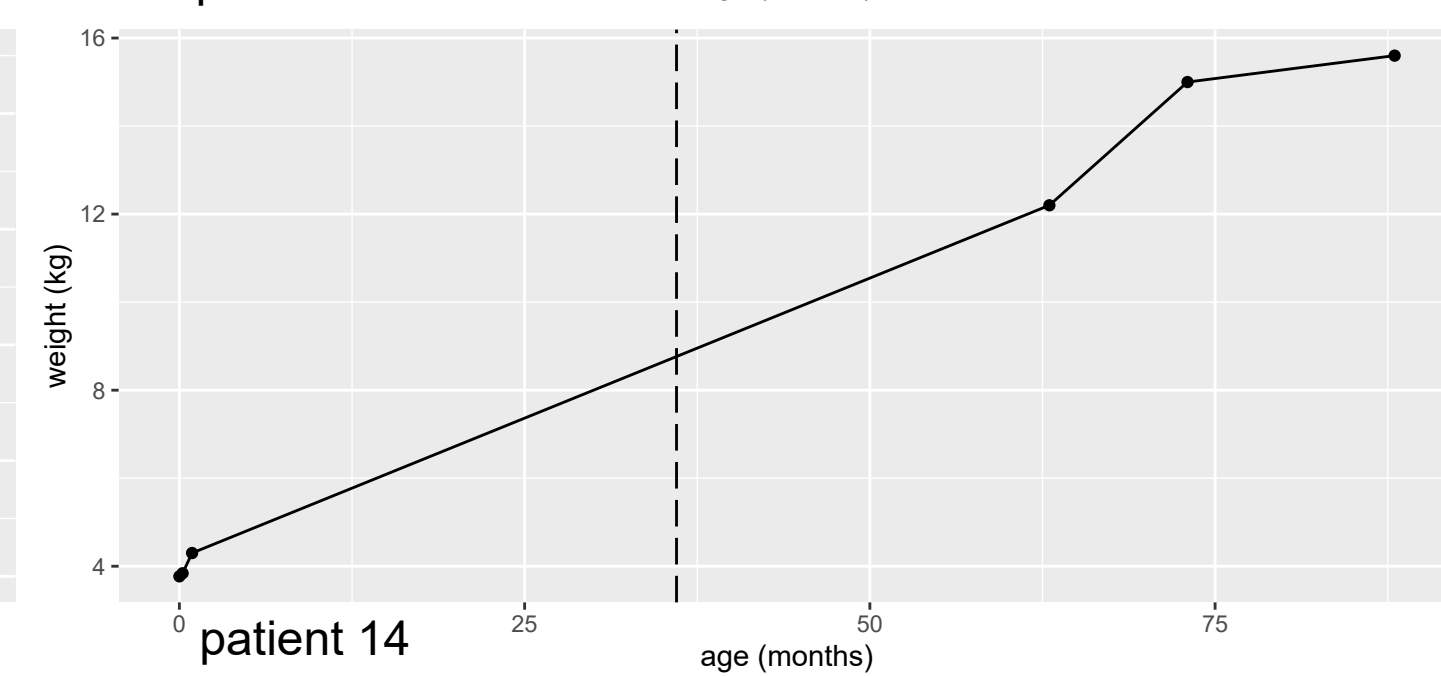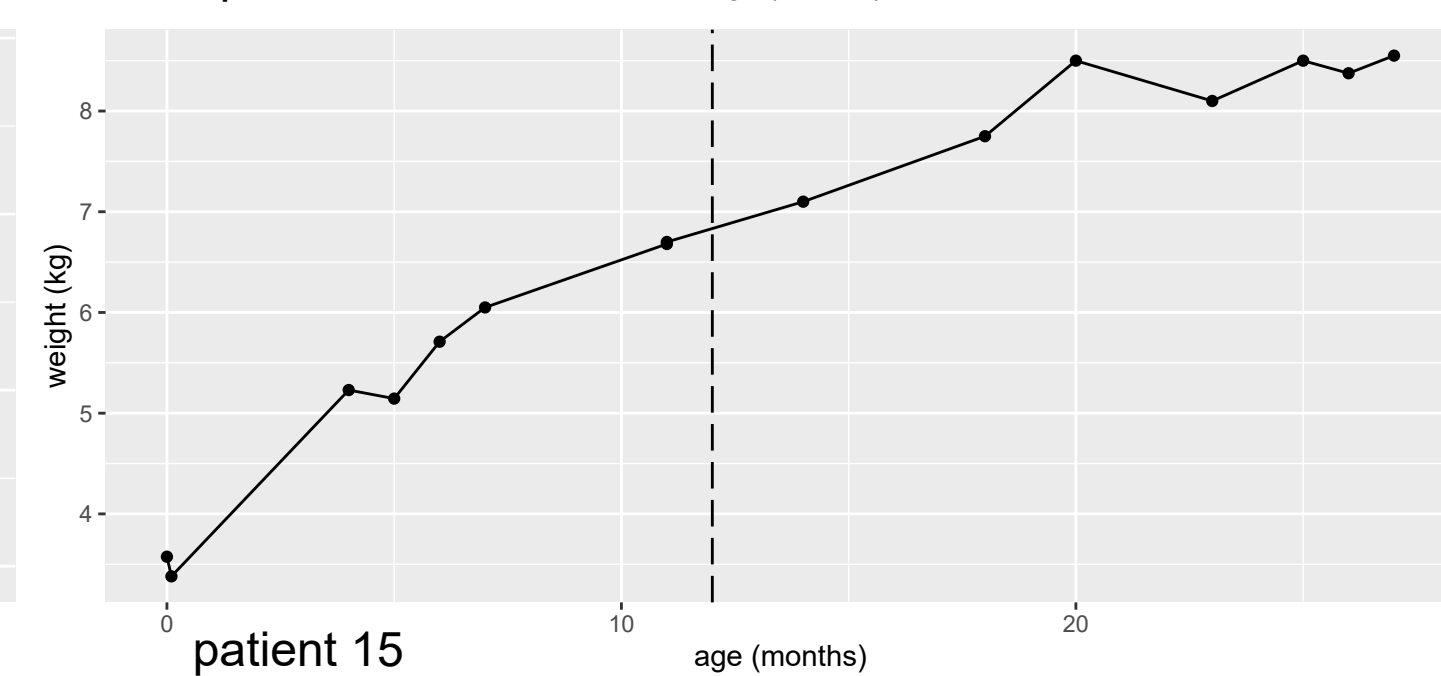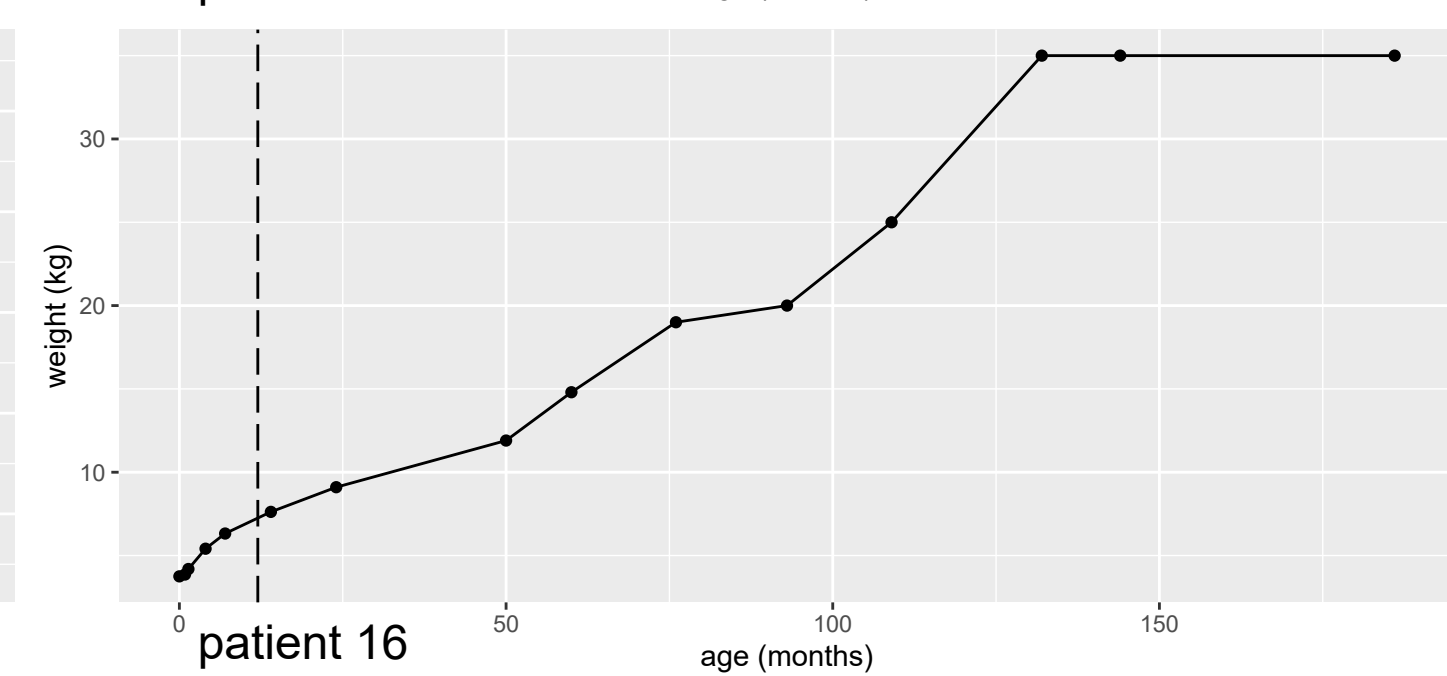

Supplement: Supplementary file 3 — Figure S2: Individual weight development for patients with early start of PEG feeding (n = 16). [file DMCN-68-82-s004.pdf]
